# Datasets on South Korean manufacturing factories’ electricity consumption and demand response participation

**DOI:** 10.1038/s41597-022-01357-8

**Published:** 2022-05-24

**Authors:** Eunjung Lee, Keon Baek, Jinho Kim

**Affiliations:** School of Energy Convergence, Gwangju Institute of Science and Technology, Gwangju, 61005 Republic of Korea

**Keywords:** Energy economics, Energy supply and demand

## Abstract

This study describes the release of electricity consumption data of some manufacturing factories located in South Korea that participate in the demand response (DR) market. The data (in kilowatt) comprise individual factories’ total power usage details that were acquired using advanced metering infrastructures. They further contain details on the manufacture types, DR participation dates, mandatory reduction capacities, and response capacities of the factories. For data acquisition, 10 manufacturing companies are representatively selected according to the process regularity and company size standard of this study. Entire datasets are newly collected and available at one-minute intervals for seven months from 1 March to 30 September 2019. These datasets can be used in a variety of ways to contribute to the functioning of power systems and markets, including the conduction of industrial load characteristic analysis for load flexibility, estimation of demand-side considerations for virtual power plant design, and determination of energy markets and incentives to achieve carbon neutrality targets at the national level.

## Abstract (summary)

**Table:** 

Measurement(s)	electricity consumption
Technology Type(s)	advanced metering infrastructure

## Background & Summary

Today, global energy and environmental conditions necessitate the widespread use of renewable energy sources for countries to achieve their carbon neutrality targets and, thereby, address climate change problems^1^. However, installing renewable energy resources without accounting for the power system reliability limitation causes system stress resulting from a supply-demand imbalance, such as from oversupply or excessive security^2^. This forces more ancillary generators in the system to stand by or promotes inefficient investment in power grid reinforcement^3^. To solve this problem, power system operators must understand the concept of load flexibility (LF). LF refers to the resources used to ensure the stable operation of the power system by facilitating dynamic changes, including increments and decrements, in demand. This includes implementing demand-side management (DSM), which changes power use patterns according to the time-series energy production characteristics of wind turbines or solar power sources to increase the application rate of renewable energy^4,5^.

The demand resources for LF are classified into industrial, commercial, and residential loads^6^. To apply the LF resources in DSM, load data at one-minute or one-hour resolution are collected for analysis, as shown in Table 1^7–18^. Further, up-to-date public data on power usage are collected to perform non-intrusive load monitoring research. They mainly include information on active power, reactive power, voltage, current, aggregated energy consumption, and appliance-level power consumption^1,5–10,13^.

**Table 1:** Summary of the details in public datasets.

Dataset	Type	Duration	Number of buildings	Sampling rate
Individual household electric power consumption dataset^7^	Residential	47 months	1	1 min
AMPds2^8^	Residential	2 years	1	1 min
Multifamily Programmable Thermostat Data^9^	Residential	3 years	79	10 min
ECO dataset^10^	Residential	8 months	6	1 Hz
DRED^11^	Residential	6 months	1	1 Hz
REDD^12^	Residential	119 days	6	1 sec
UK-DALE^13^	Residential	2.5 years	5	1 min
ENERTALK^14^	Residential	29–122 days	22	15 Hz
100 EnerNOC Commercial Buildings^15^	Commercial	1 year	100	15 min
CU-BEMS^16^	Commercial	18 months	2	1 min
Industrial machines dataset for electrical load disaggregation^17^	Industrial	111 days	8	1 sec
Food and paper industries^18^	Industrial	3 years	3	1 h

However, although most of the DSM capacity for LF is met by industrial loads, there are quite a few obstacles to the acquisition of industrial demand data. In a competitive industrial environment, the data disclosure of industrial loads is prohibited since such data are considered a trade secret because a manufacturing plant’s electricity consumption data can be used to infer the company’s sales. To the best of the authors’ knowledge, investigations on manufacturing factories’ load data remain limited; only two studies require special mention in this respect: an investigation on the machine-level load data of a paper manufacturing factory in Brazil^17^ and an examination of the normalized electricity consumption data of food and paper industries^18^.

In this study, the authors acquire data from volunteered industrial factories and analyze their characteristics to evaluate demand response (DR) availability of Korean industrial demands for securing power system and market flexibility. Furthermore, a market system is being designed to encourage factories to participate as LF resources.

The authors collect electricity consumption data from manufacturing factories in South Korea by using communication systems, including the advanced metering infrastructure (AMI). These factories participate in the DR market through DSM. Accordingly, the resulting dataset is unique and potentially a valuable consideration in several analyses, including.

- Expected locational DR capacity estimation by statistically estimating customer baseline load (CBL) and participation amount of each industrial sector.
- Estimation of hourly LF by analyzing industrial demand consumption patterns.
- Consideration of demand-side utilization in virtual power plants.
- Design of the LF market and incentive price.

## Methods

The load aggregators performing brokerage transactions in the DR market are authorized to collect electricity usage information from the system operator through the AMI for DSM. In this study, the authors first introduce international and Korean demand response programs in detail. Subsequently, they describe a novel communication system in which a load aggregator collects relevant data through the AMI and finally classify the industrial demand data collected from the factories participating in DR programs by manufacture type.

### Demand response programs

**Fig. 1:**
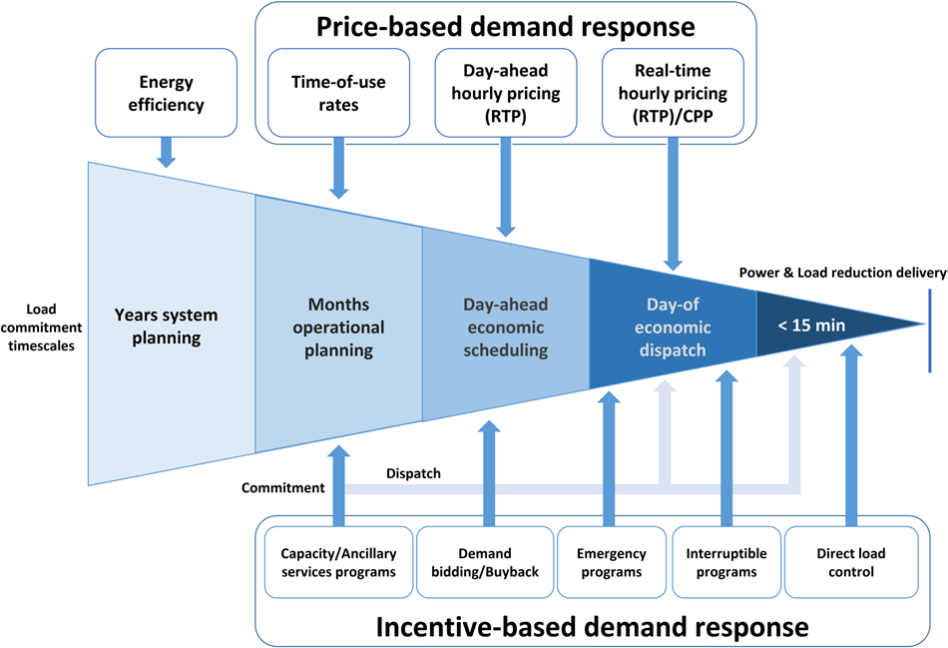
Role of DR in electricity system planning and operation.

DR is defined as a tariff or a program established to motivate changes in electric use by end-use customers in response to changes in the price of electricity over time or to give incentive payments designed to induce lower electricity use at times of high market prices or when grid reliability is jeopardized^19^. It is classified into price-based DR for economic operational purposes and intensive-based DR for system security purposes. Figure 1 illustrates DR programs included in the planning and operation of power system in detail. In DR programs, the participation performance of resources is evaluated based on CBL estimation^19^. In general, the average demand usage of past days without participating in DR is used in calculating CBL. Table 2 describes DR services of independent system operators (ISOs) in the US, which are internationally benchemarked^20–25^.

**Table 2:** Summary of the details of CBL evaluation methods for ISO in the US.

ISO	Service	CBL evaluation method	Adjustment option	Description
MISO^20^	Contingency reserve service	Meter-before	X	Power consumption in the 10-second interval prior to the start of the DR participation time
	Regulation reserve service	Meter-before	X	Power consumption for the 5-minute interval preceding the start of the DR participation time
	Energy	Average	O	Average power consumption for 10 days out of the past 10 days excluding holidays and weekends
NYISO^21,22^	Emergency and day-ahead DR	Average	X	Average power consumption for lowest 5 days out of the past 10 days excluding holidays and weekends
PJM^23,24^	Economic, pre-emergency and emergency DR	Average	O	Average of 3 hours prior to the DR participation time and 2 hours after the DR participation time
		Average	O	Average power consumption for 5 days out of the past 5 days excluding holidays and weekends
		Matching day pair	O	Average power consumption for 3 days most similar with DR participation day
ERCOT^25^	Emergency response service	Regression	O	Baseline estimation based on the correlation model of power consumption for weather condition on the day and preceding days, the type of day, and daylight
		Average	O	Average power consumption for 8 days out of the past 10 days excluding highest, lowest consumption days
		Average	O	Average power consumption for 20 days out of the past 20 days excluding holidays and weekends
		Matching day pair	O	Average power consumption for 10 days most similar with DR participation day
		Meter-before	X	Power consumption for the immediately preceding time

Korean DR market consists of six programs depending on the purpose as shown in Table 3^26^. In recent years, along with traditional DR programs, they expanded to mitigate environmental issues, including fine dust problems and supply/demand balance due to rapid renewable energy penetration. Participants are restricted from entering the market depending on the type and capacity of resources they have. Table 4 describes Korean ISO’s DR services in detail^26^.

**Table 3:** Summary of the details of the DR program in South Korea.

DR program		Purpose
Voluntary DR	Economic DR	Power supply cost reduction by being participated in the power market in the same way as conventional generators
	Peak demand DR	Reserve capacity securement in accordance with excess of forecasted demand compared with baseline
	Fine dust DR	Reduction of power supply cost and fine dust
Reliability DR		Substitution of new power generator construction depending on demand reduction during forecasted emergency periods
Frequency DR		Frequency drop prevention below stability operating standard
Reverse DR		Reduction of renewable energy curtailment

**Table 4:** Summary of the details of CBL evaluation methods in South Korea.

CBL evaluation method	DR program	Description
Max 4 of 5	Standard DR	Average power consumption for top 4 days out of the past 5 days excluding holidays and weekends
Mid 6 of 10	Standard DR	Average power consumption for 6 days out of the past 10 days excluding highest and lowest consumption 2 days
Mid 4 of 6	Reverse DR (weekdays) and residential DR	Average power consumption for 4 days out of the past 6 days excluding highest and lowest consumption days
Mid 8 of 10	Residential DR	Average power consumption for 8 days out of the past 10 days excluding highest and lowest consumption days
Past 10 minute	Frequency DR	Sum of the 1-minute interval power consumption for 10 minutes prior to the start of the DR participation time multiplied by 6
H-mid 4 of 6	Reverse DR (weekends and holiday)	Average power consumption for 4 days out of the past 6 days (holidays and weekends), excluding highest and lowest consumption days

### Monitoring set-up

**Fig. 2:**
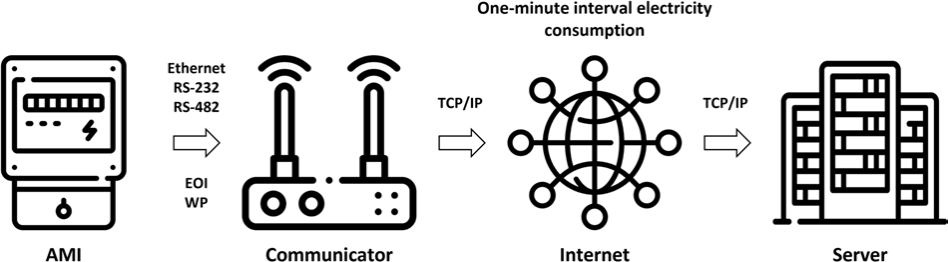
Overall hardware communication network used in the study. EOI, end of interval; IP, Internet Protocol; TCP, Transmission Control Protocol; WP, watthour pulse.

In the proposed communication system, watthour pulse (WP) and end-of-interval (EOI) signals are received in one-minute units through the AMI’s photocoupler, which is installed to charge electricity bills to the manufacturing company. The WP-based wattage data are synchronized with the EOI signal and delivered to the server in real-time. Further, the system involves storing the process of monitoring data for a short period to improve data acquisition quality. When data delivery fails, the communication system performs a resending the stored data to the server. After a certain number of retries fail, the data is extinguished by storage period expiration. The well-collected data are backed-up every 30 days. To upload the data to the server, one can select the interface from among Ethernet, RS-232, and RS-482 ports according to the communication environment. Figure 2 illustrates the overall hardware communication network design.

### Industrial demand data classification

In Korea, the manufacturing industry is classified into 40 industries. Among them, 10 industries, namely petrochemical, fine chemical, cement, steel, forging, food, paper, metal, electricity/electronics, and textile, mainly participate in the DR market and function as ancillary service resources. The number of their companies account for 44.92% of all industries. The authors selected five representative types which account for 48.36% of the aforementioned 10 manufacturing factories: cement, forge, metal, paper, and steel. Only 11.59% of the companies included in the types are actually participating in the DR program. Therefore, it is expected that they still have high potential that can be utilized as LF resources^27^.

**Fig. 3:**
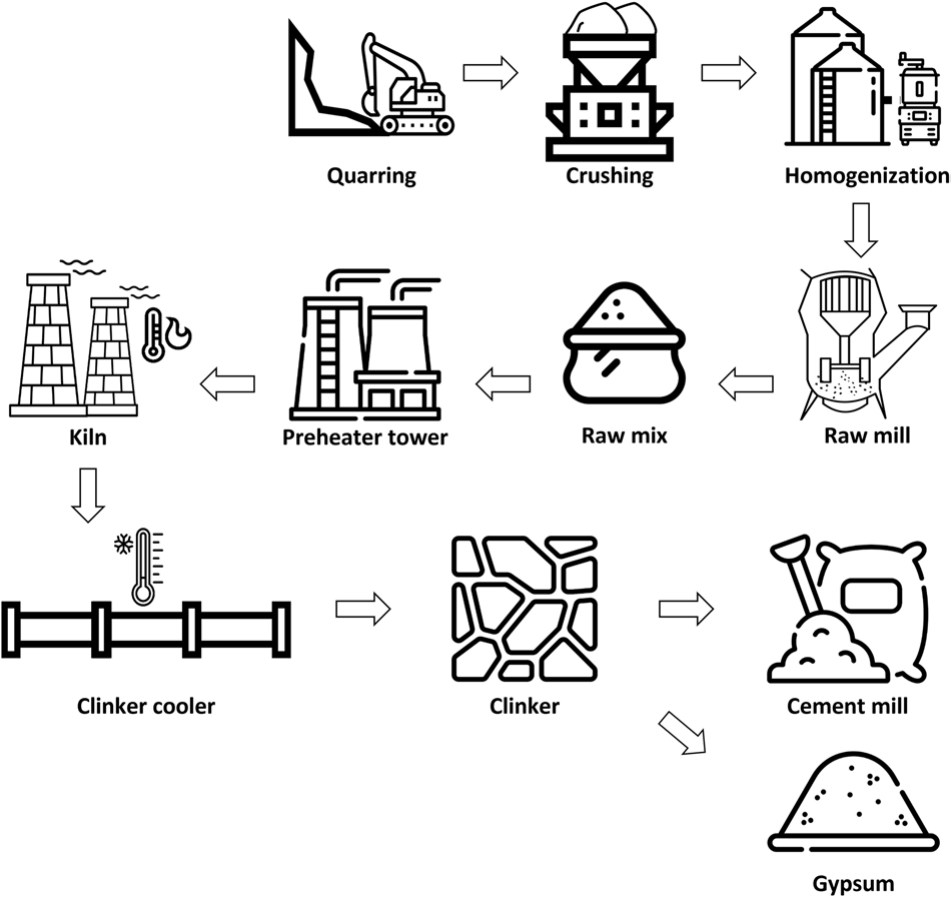
Cement manufacturing process.

**Fig. 4:**
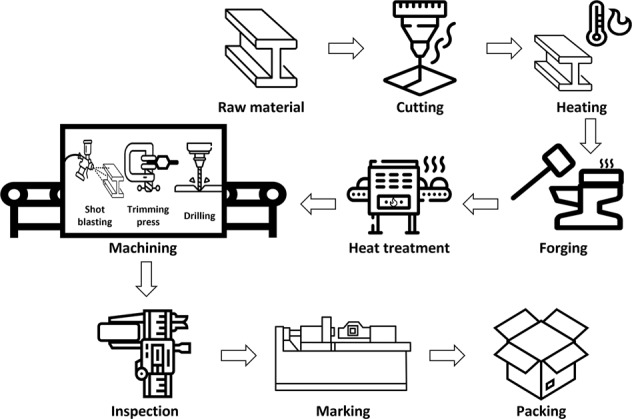
Forging process.

**Fig. 5:**
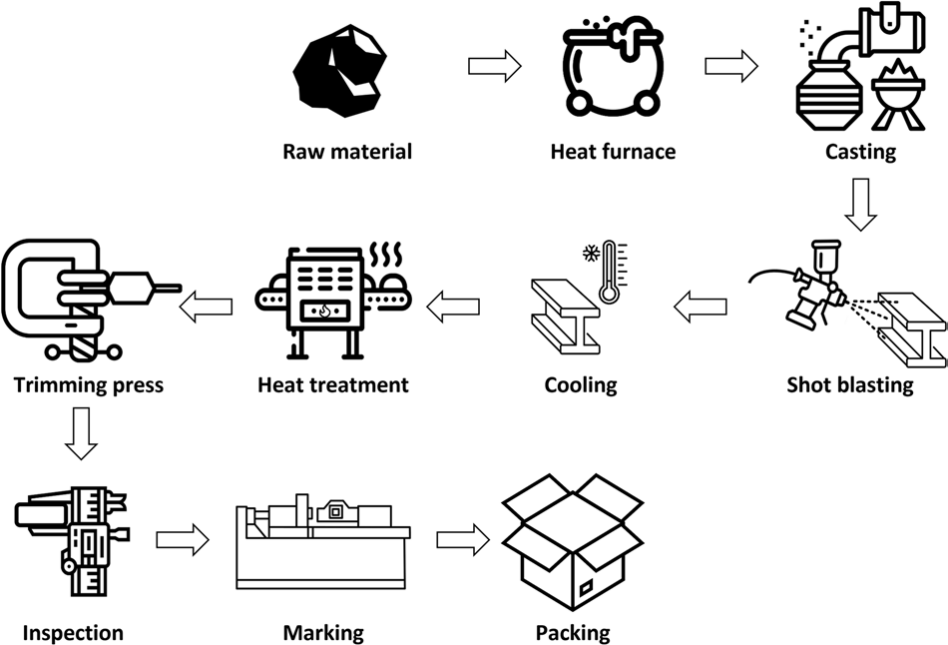
Metal casting process.

**Fig. 6:**
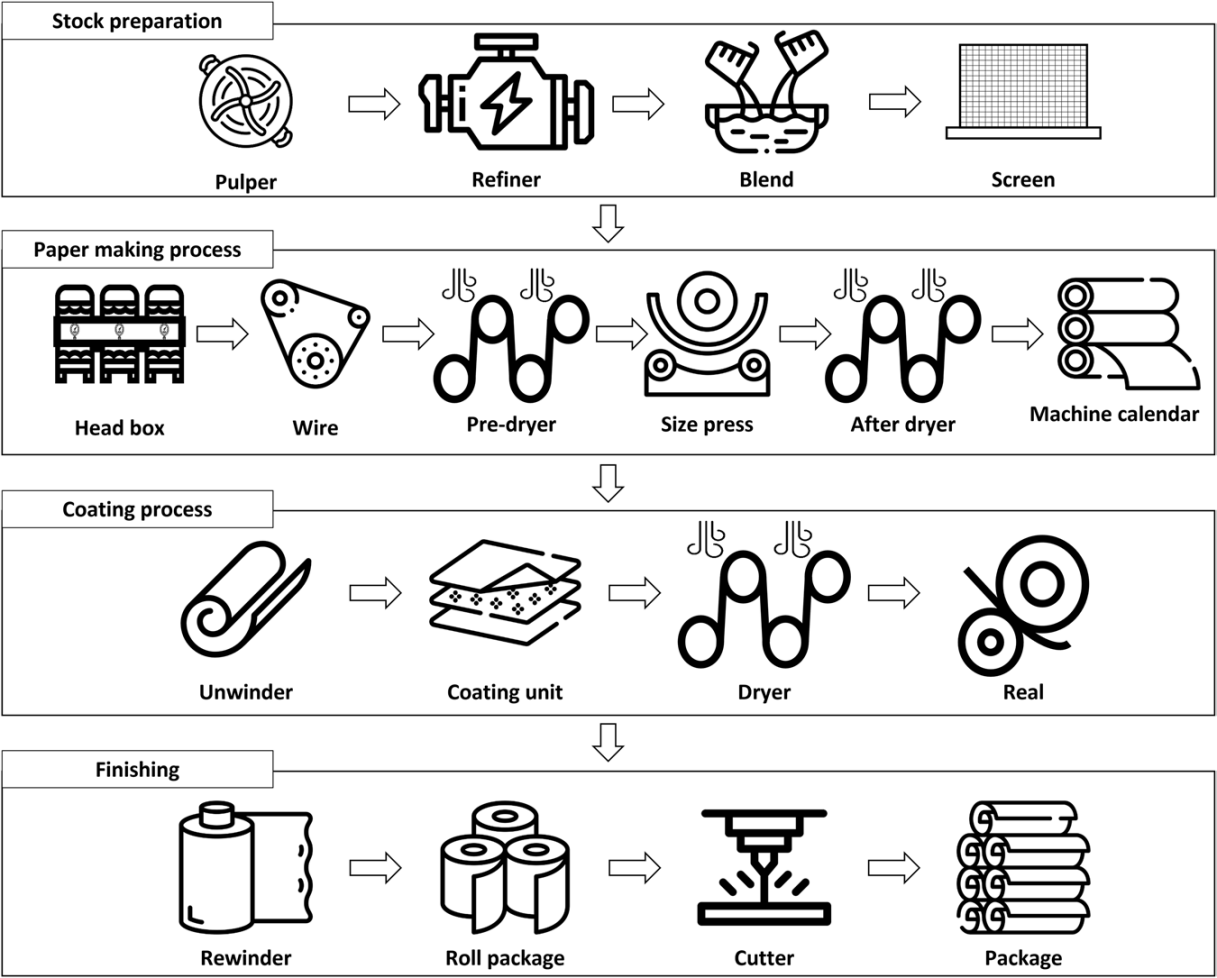
Paper manufacturing process.

**Fig. 7:**
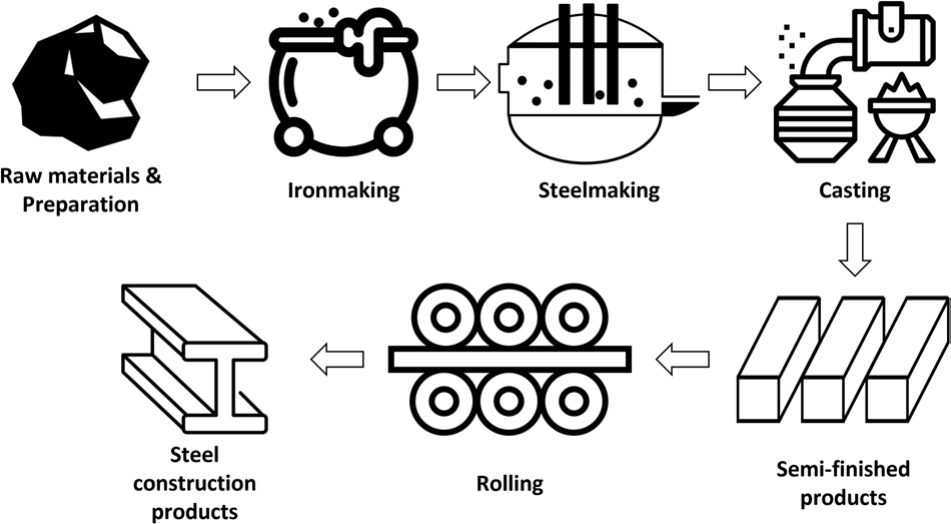
Steel manufacturing process.

Data from 20 volunteer factories with data disclosure agreements were obtained. Finally, 10 factories with regular manufacturing processes and their company sizes (e.g., number of employees, sales, and manufacturing scales) were selected in this study. Figures 3–7 illustrate the five representative manufacturing processes. To maintain information security, the company name and factory location are not disclosed in this paper, and net power consumptions without normalization are mentioned to preserve data originality. This study presents the data measured for seven months from 1 March 2019 to 30 September 2019. During the measurement period, a DR was issued twice; Table 5 depicts the date and time of DR participation, mandatory reduction capacity, and response capacity of each factory for the load aggregator’s transaction.

**Table 5:** DR market participation records of manufacturing factories.

Manufacturing factory	DR participation date(s)	Mandatory reduction capacity (kW)	Responded capacity (kW)
Metal 1	18:00–19:00, 13 June 2019	8000	8777
Metal 2	17:00–20:00, 15 May 2019 16:00–17:00, 13 June 2019	24000/24000/24000 24000	25737/25874/26822 24279
Metal 3	18:00–19:00, 13 June 2019	8000	10727
Forge 1	18:00–19:00, 13 June 2019	6000	4440
Forge 2	18:00–19:00, 13 June 2019	4000	9
Steel 1	18:00–19:00, 13 June 2019	4000	3925
Steel 2	18:00–19:00, 13 June 2019	60000	195415
Cement 1	18:00–19:00, 13 June 2019	45000	51198
Cement 2	18:00–19:00, 13 June 2019	13000	18999
Paper	18:00–19:00, 13 June 2019	25000	12510

DR, demand response.

## Data Records

The entire dataset comprises 10 comma-separated value (CSV) files^28^, summarised in Table 6. As mentioned earlier, the total electricity consumption (kW) of each factory was measured in this study. The CSV files of each factory have 308160 rows, including N/A spaces and outliers, which indicate one-minute-interval data (1440 data points/day) for 214 days during the 7-month data collection period in 2019. Since the method of preprocessing data is selected and applied according to various research purposes, the authors provided raw data for reuse without preprocessing. Each file has two columns: one indicates time information (in the YYYY-MM-DD hh:mm format), while the other indicates the factory’s real-time electricity consumption. For better reuse, the Korean system load data file of the same period is provided together^28^. The dataset has been made publicly available under the creative commons license CC BY 4.0 hosted on the figshare repository.

**Table 6:** Summary of manufacturing factories’ dataset file names.

Manufacturing factory	Name	The number of data	Data periods
Cement 1	Cement_1.csv	306941	2019–03–01~2019–09–30
Cement 2	Cement_2.csv	307475	2019–03–01~2019–09–30
Forge 1	Forge_1.csv	306656	2019–03–01~2019–09–30
Forge 2	Forge_2.csv	308029	2019-03-01~2019-09-30
Metal 1	Metal_1.csv	208154	2019-03-01~2019-09-30
Metal 2	Metal_2.csv	276938	2019–03–01~2019–09–30
Metal 3	Metal_3.csv	307566	2019–03–01~2019–09–30
Paper	Paper.csv	308158	2019–03–01~2019–09–30
Steel 1	Steel_1.csv	303501	2019–03–01~2019–09–30
Steel 2	Steel_2.csv	308160	2019–03–01~2019–09–30

## Technical Validation

This section discusses the visualization of data to clarify the quality of the dataset, which includes missing data, outliers, and weekly pattern plots. The missing data plot and outlier information indicate the availability of minute details on the electricity consumption of each factory, whereas the weekly pattern plots provide the characteristic insights into power consumption according to the manufacturing type and working/non-working date conditions. The summary of manufacturing factories’ dataset statistics is described as shown in Table 7.

**Table 7:** Summary of manufacturing factories’ dataset statistics.

Manufacturing factory	Mean	Standard deviation	0^th^ percentile	25^th^ percentile	50^th^ percentile	75^th^ percentile	100^th^ percentile
Cement 1	1095	293	0	1008	1187	1277	2854
Cement 2	530	100	0	470	549	594	7482
Forge 1	57	48	0	2	81	102	188
Forge 2	54	35	0	6	67	82.6	119
Metal 1	124	73	6	29	154	190	259
Metal 2	369	224	0	73	451	543	786
Metal 3	111	80	0	26	115	179	294
Paper	480	88	0	420	521	554	857
Steel 1	47	36	0	13	35	78	131
Steel 2	7375	2705	0	5310	7872	9522	14966

### Missing data

**Fig. 8:**
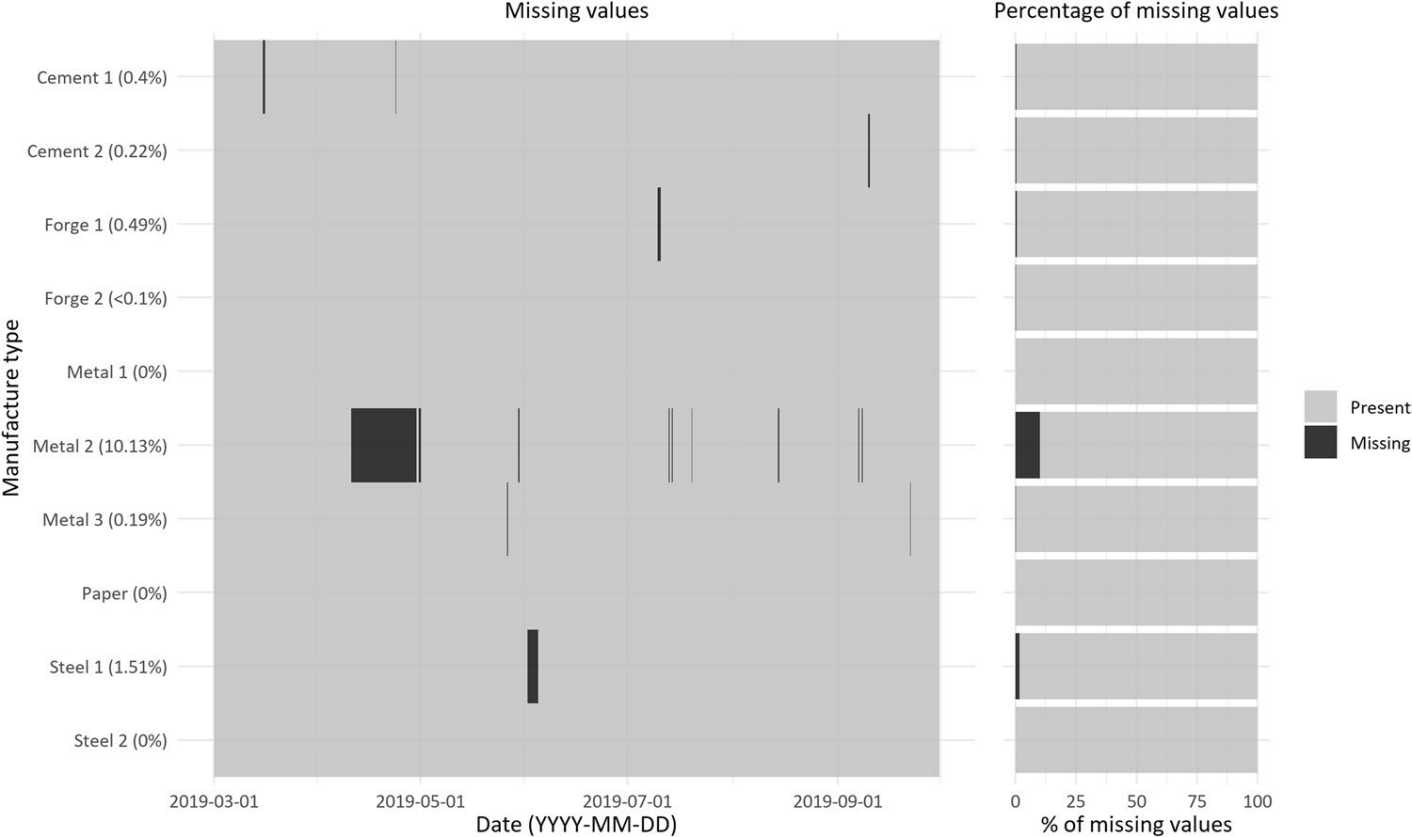
Missing electricity consumption data of 10 manufacturing factories; the missing data are indicated using black lines.

Figure 8 illustrates the missing electricity consumption data of 10 factories. The missing data plot for the entire data collection period (where the missing data are indicated using black lines) is shown on the left side of the figure. Further, the horizontal bars on the right visually represent the percentage of missing data over the study period. The manufacturing factories have an average data availability of 98.7%. An exception is the Metal 2 factory, whose missing data rate is more than 10% due to data collection errors in April 2019. Data with a 20% or less missing rate guarantees quality through missing data imputation^29^. The approach for time-series missing data imputation provided in this study is classified mainly into five categories: deletion, neighbor-based, regression-based, multi-layer-perceptron-based, and deep-learning-based approaches. The description and practical methods of each approach were reviewed in detail as shown in Table 8^30–40^.

**Table 8:** Summary of missing data imputation methods for time series data.

Approach	Description	Method
Deletion^30,31^	Elimination of observations with missing values in raw data	Listwise deletion and pairwise deletion
Neighbour based^32,33^	Missing data imputation through neighbours identified by the clustering method	KNN and DBSCAN
Regression based^34,35^	Missing data prediction by modelling correlations between a dependent variable and independent variables based on historical data	AR, ARX, and ARIMA
Multi-layer perceptron based^36,37^	Missing data estimation by designing a model minimizing the loss function of fully connected network	NLP and ANN
Deep learning based^38–40^	Missing data prediction by designing network including information over time	RNN and GRU

### Outliers

**Fig. 9:**
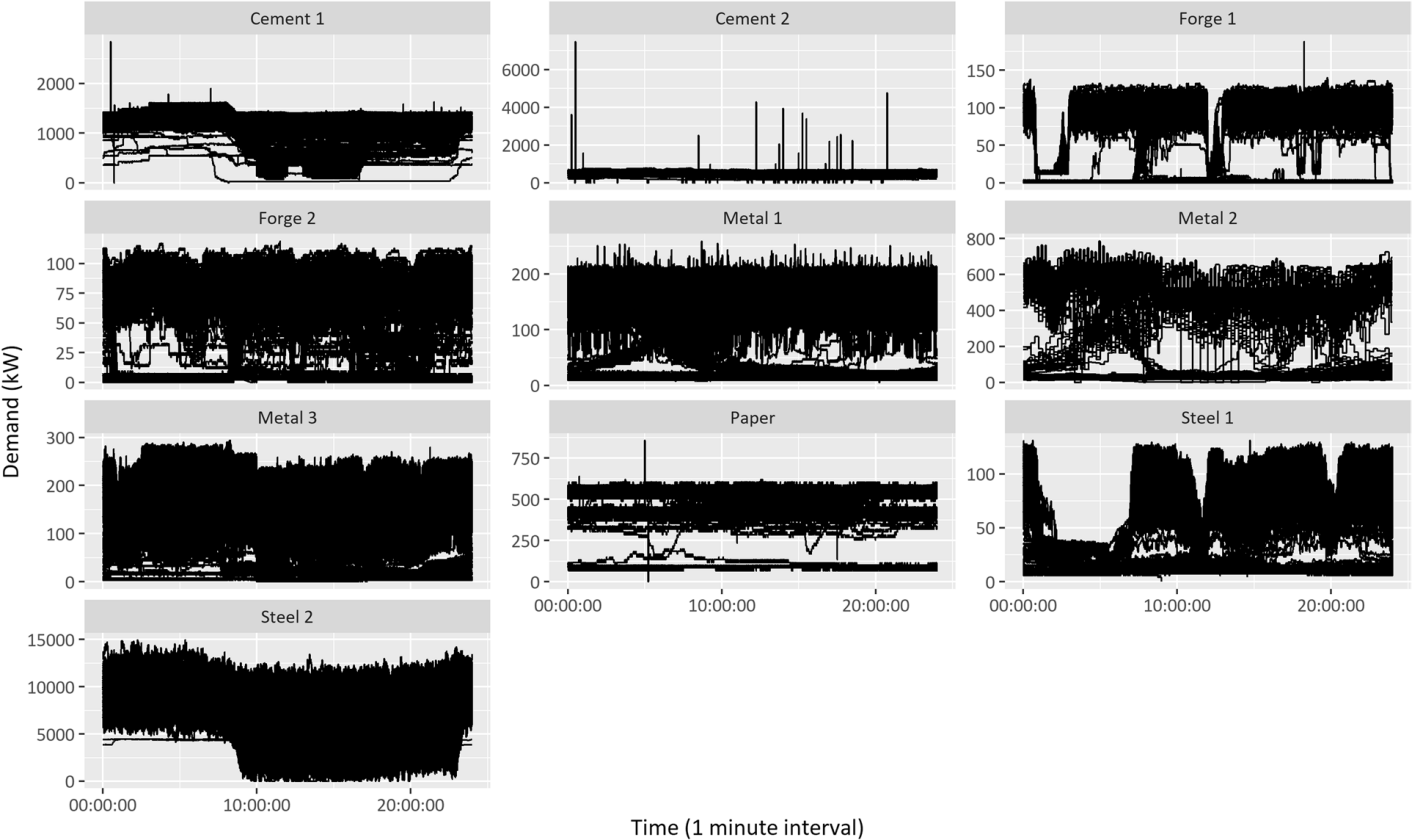
Electricity consumption daily profiles of 10 manufacturing factories during data collection periods.

Figure 9 illustrates the 10 factories’ daily electricity consumption profiles during data collection periods. As an index for outlier detection, the interquartile range (IQR) of the box plot was considered. As a result of extracting data located outside the range of 3 sigma of the normal distribution from each demand data, 4, 38, and 1 outlier were detected in Cement 1, Cement 2, and Paper, respectively. The approach for time-series outlier data detection provided in this study is classified into four categories: statistical, unsupervised discriminative, unsupervised parametric, and supervised approaches. The description and practical methods of each approach were reviewed in detail as shown in Table 9^41–50^. Accordingly, the authors propose to scale and utilize the raw data according to the research purpose.

**Table 9:** Summary of outlier detection methods for time series data.

Approach	Description	Method
Statistical approach^41–43^	Outlier detection through a function describing the relationships between a dependent variable and independent variables based on historical data	ARMA, ARIMA, VARIMA, and EWMA
Unsupervised discriminative approach^44,45^	Outlier detection through similarity measurement based on clustering method	K-means, SOM
Unsupervised parametric approach^46–48^	Outlier detection through probabilistic model about state or value over time	HMMs
Supervised approach^49,50^	Outlier detection through a model trained with labelled data	SVM

### Weekly patterns

**Fig. 10:**
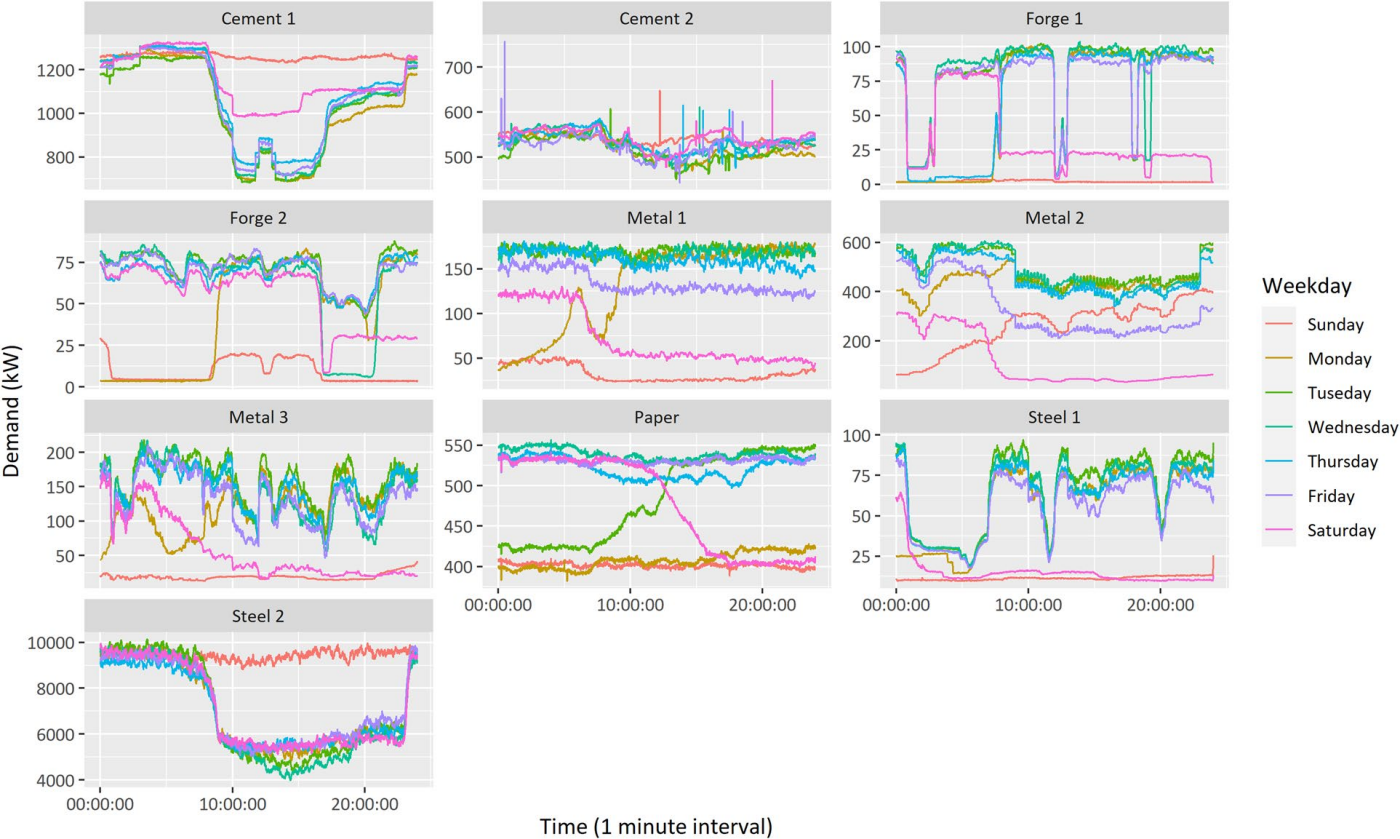
Weekly electricity consumption patterns of 10 manufacturing factories.

Figure 10 shows the 10 factories’ weekly electricity consumption patterns, obtained by averaging the electricity consumption during the data collection period by day of the week. Each factory reveals approximate periodicity according to its own manufacturing process. The factories that implemented automated processes (Steel 2, Cement 1, and Cement 2) recorded a steady electricity use even on non-working days. The factories’ electricity consumption varied according to their size; for example, employees, sales, and production scale. In particular, factories with high electricity usage (Metal 2, Steel 2, and Cement 1) tended to avoid operating on time intervals with high electricity rates. Despite the limitation of the 7-month acquisition period, the characteristics of weekly demand usage were strongly confirmed.

**Fig. 11:**
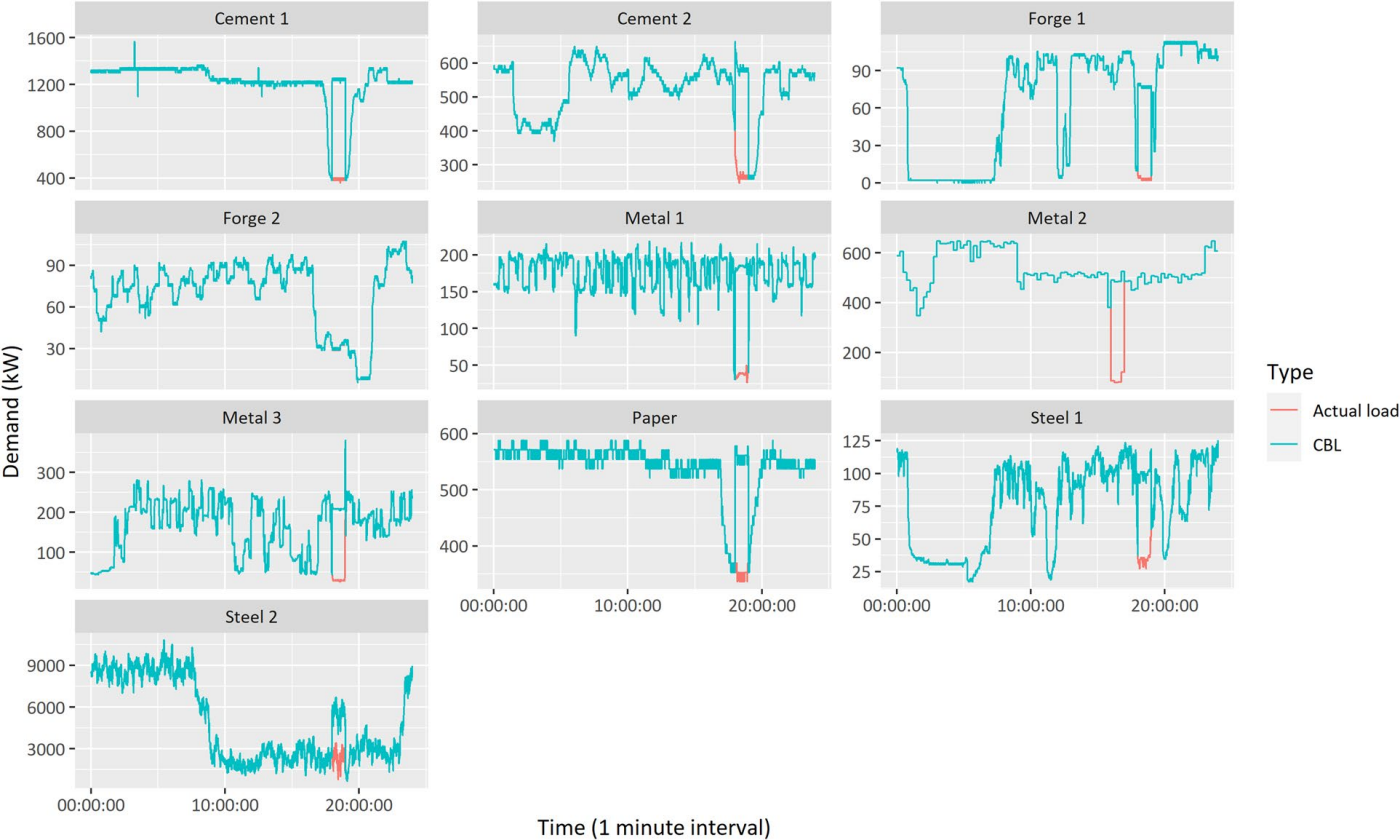
Manufacturing factories’ electricity consumption profiles at the demand response participation day (13 June 2019); cyan lines indicate customer baseline load (CBL), and red lines indicate the actual load.

Figure 11 provides the factories’ electricity consumption profiles at the DR participation day (13 June 2019), which confirm the factories’ responded capacities. The capacity is calculated as the difference between the CBL (denoted using cyan lines in Fig. 11) and the actual load (denoted using red lines). The CBL is a general standard used for settlement in national DR markets. In this study, the factories’ average power consumption in the same time for four out of the past five days, excluding holidays, is considered the CBL. As additional information, Fig. 12 indicates the power system demand profile at the DR participation days (15 May and 13 June 2019) in South Korea.

**Fig. 12:**
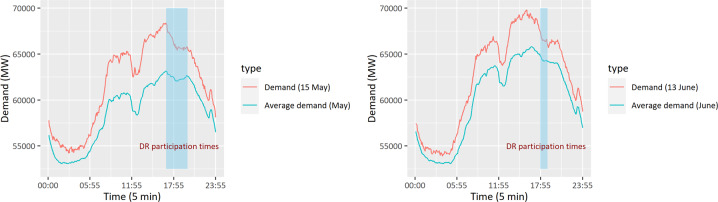
Power system demand profiles; cyan lines indicate average demand for the month, including the demand response participation days (15 May and 13 June 2019), and red lines indicate demand at the participation days.

## Data Availability

The code implementation was done in R 4.0.5 using R studio. The scripts to perform data visualization are available in^28^.
